# MicroRNA-326-5p enhances therapeutic potential of endothelial progenitor cells for myocardial infarction

**DOI:** 10.1186/s13287-019-1413-8

**Published:** 2019-11-15

**Authors:** Xiaoting Li, Xiang Xue, Yuejun Sun, Lei Chen, Ting Zhao, Wentao Yang, Yongbing Chen, Zhiwei Zhang

**Affiliations:** 1 Department of Cardiology, The Second Affiliated Hospital of Soochow University, No.1055, Sanxiang Road, Suzhou, 215004 China; 2 Department of Cardiothoracic Surgery, The Second Affiliated Hospital of Soochow University, No.1055, Sanxiang Road, Suzhou, 215004 China; 3 Department of Pathology, Affiliated Jiangyin Hospital of Southeast University Medical College, Jiangyin, 214400 Jiangsu China

**Keywords:** Mir-326-5p, Endothelial progenitor cells, Myocardial infarction, Angiogenesis

## Abstract

**Background:**

Our study sought to investigate the therapeutic effects and mechanisms of miR-326-5p-overexpressing endothelial progenitor cells (EPCs) on acute myocardial infarction (AMI).

**Methods:**

Mouse EPCs were isolated, purified, and identified by flow cytometry and uptake of DiI-ac-LDL. The target gene of miR-326-5p was predicted using target prediction algorithms and verified by dual-luciferase reporter assay, RT-qPCR, and Western blot. After EPCs were transfected with the agomir or antagomir of miR-326-5p, tube formation assay and Matrigel plug angiogenesis assay were conducted in four groups (NC, miR-326-5p agomir, miR-326-5p antagomir, and miR-326-5p agomir+Wnt1 agonist). In addition, a mouse model of MI was established and treated with the injection of miR-326-5p-EPCs, miR-326-5p-EPCs+ Wnt1 agonist, EPCs-NC, or PBS/control into the peri-infarcted myocardium. Subsequently, cardiac function was monitored by echocardiography at 7 and 28 days postoperatively. Finally, the infarcted hearts were collected at 28 days, and the size of myocardial infarction was measured by Masson’s trichrome staining and the neovascularization in the peri-infarcted area was examined through immunofluorescence staining.

**Results:**

Luciferase reporter assay indicated that Wnt1 was a direct target of miR-326-5p. Using RT-qPCR and Western blot analysis, we further demonstrated that the expression level of Wnt1 was negatively correlated with miR-326-5p expression in EPCs. Both in vitro study of tube formation assay and in vivo investigation of subcutaneous Matrigel plug assay revealed that the miR-326-5p agomir could significantly enhance the angiogenic capacity of EPCs, and this effect was partially inhibited by Wnt1 agonist. Meanwhile, miR-326-5p antagomir could obviously reduce the the angiogenic capacity of EPCs in vivo compared with that in the NC group. Moreover, the transplantation of miR-326-5p-overexpressing EPCs in the ischemic hearts of mice significantly enhanced the angiogenesis in the peri-infarcted zone and improved the cardiac function. However, the enhanced capacity of angiogenesis of miR-326-5p-overexpressing EPCs was remarkably neutralized by Wnt1 agonist, accompanied by the decreased improvement in cardiac function.

**Conclusion:**

miR-326-5p significantly enhanced the angiogenic capacity of EPCs. Transplantation of miR-326-5p-overexpressing EPCs improved cardiac function for AMI therapy, which can be a novel strategy for enhancing therapeutic angiogenesis in ischemic heart diseases.

## Background

Ischemic heart disease (IHD), specifically acute myocardial infarction (AMI), is the leading cause of morbidity and mortality in the world [1]. Ischemia and hypoxia are the main pathological process in MI, subsequently causing myocardial apoptosis, inflammation, arrhythmia, myocardial fibrosis, cardiac remodeling, and heart failure [2]. Therefore, effective revascularization is of utmost importance. Previous studies have reported that several types of stem cells safely and efficiently improved the neovascularization in the local ischemic region [3], especially endothelial progenitor cells (EPCs) [4, 5].

Asahara et al. [6] found that the bone marrow origin of EPCs greatly contributed to the postnatal physiological and pathological neovascularization. Ander et al. further confirmed in the PROCELL study [7] that EPCs could be significantly mobilized from the bone marrow into the peripheral circulation after AMI, which was positively correlated with the level of vascular cell adhesion molecule (VCAM)-1 in plasma. Then, circulating EPCs intensively home to and incorporate into the target of ischemic heart [8]. Finally, EPCs differentiate into mature endothelial cells and initiate the repair process of neovascularization [9]. Therefore, direct transplantation of EPCs into the ischemic myocardium has been conducted and exhibited cardiac protective effects, including augmenting neovascularization, decreasing inflammatory apoptosis, and improving cardiac function [4, 10].

Particularly, microRNAs (miRNAs), a class of small (18–22 nucleotides) single-stranded noncoding RNAs, have been identified as novel regulators involved in the angiogenesis, differentiation of progenitor cell, and heart development. Therefore, they are promising potential therapeutic targets for the treatment of heart diseases [11–13]. Regarding the mobilized EPCs from the bone marrow, Templin et al. [14] further demonstrated that miR-378 and let-7b in CD34^+^ EPCs of patients with ST-segment-elevation myocardial infarction (STEMI) were significantly upregulated, and in particular, miR-378 could promote the angiogenic capacity of CD34^+^ EPCs in vivo. For the therapeutic MI, Stefan’s lab [15] revealed that inhibiting miR-377 in CD34^+^ bone marrow-derived cells could facilitate angiogenesis in the ischemic myocardium and further improve cardiac function and inhibit myocardial remodeling. Comprehensibly, many miRNAs might hold the potential of regulatory angiogenesis in EPCs, which is beneficial for cardiac repair. In this study, we sought to investigate the effects of miR-326-5p on the function of EPCs for the treatment of ischemic myocardial disease. We conducted gain- and loss-of-function investigations of miR-326-5p to examine the angiogenic effects on EPCs and the functional cardiac repair of miR-326-5p-overexpressing EPCs for infarcted hearts. We demonstrate for the first time that EPCs treated with miR-326-5p could significantly improve cell angiogenic capacity and functional recovery in a murine model of myocardial infarction.

## Methods

### Animals

All mice were purchased from the Laboratory Animal Center of Nanjing University (Nanjing, China). The experimental procedures were approved by the institutional animal ethics guidelines for the Care and Use of Research Animals established by Soochow University (Suzhou, China). Special efforts were made to minimize animal suffering.

### Isolation, culture, and characterization of mouse EPCs

Mouse EPCs were isolated and cultured as described previously [16]. Briefly, the bone marrow was extracted from the femora and tibiae of C57BL/6 mice of 4 weeks old. Bone marrow-mononuclear cells (BM-MNCs) were further layered by density gradient centrifugation (Histopaque 1083, Sigma-Aldrich, USA). After being washed twice, cells were seeded onto a culture dish and cultured in EGM-2 MV (Lonza, Switzerland) supplemented with 5% fetal bovine serum (FBS). Then, the cells were incubated at 37 °C in a humidified atmosphere containing 5% CO_2_. The culture medium was refreshed every 48 h. When grown to 90% confluence, cells were harvested with 0.25% trypsin (Sigma-Aldrich, USA) and passaged continuously. The phenotypes of EPCs were examined by flow cytometry using antibodies against mouse CD11b, CD31, CD45, CD133, VE-cadherin, and Flk-1, respectively (Sigma, USA). DiI-ac-LDL uptake assay was conducted to further investigate the characteristics of EPCs.

### Target gene prediction and dual-luciferase reporter assay

TargetScan (http://targetscan.org), microRNA.org (http://www.mirtoolsgallery.org/miRToolsGallery/node/1055), and mirWALK (http://zmf.umm.uni-heidelberg.de/apps/zmf/mirwalk2/) were used to predict the target genes of miR-326-5p, and miR-326-5p was a candidate of regulators for Wnt1. The PCR product of the Wnt1 3′-UTR full-length fragment or mutant fragment was double-digested and then ligated into the pmirGLO Vector. After sequencing, the plasmid was designated as pmirGLO-Wnt1-WT and pmirGLO-Wnt1-MUT. Plasmids with WT or MUT 3′-UTR DNA sequences were transfected with miR-326-5p agomir (100 nM; Sangon Biotech, China) or negative control agomir into HEK293T cells (ATCC, USA). After cultivating at 37 °C for 24 h, cells were examined using the dual-luciferase assay system (Promega, USA) according to the manufacturer’s instructions.

### Quantitative real-time PCR assay

Total RNA was isolated from EPCs using TRIZOL (Invitrogen, USA), and reverse transcription was performed using the microRNA reverse transcription system (GenePharma, China) or the PrimeScript RT reagent kit (TAKARA, Japan). The expression level of miR-326-5p was analyzed by SYBR Green assay kit (TAKARA, Japan) with U6 as control. For expression analysis of Wnt1 mRNA, RNA was quantified using SYBR PCR master mix in the ABI Step One-Plus Detection system (Applied Biosystems, USA) according to the manufacturer’s instructions. GAPDH was used as an internal control. Quantitative primer sequences for Wnt1 were 5′-CCACCTCTTCGGCAAGATCGTCAA-3′ (forward) and 5′-GTGGCATTTGCACTCTTGGCGCAT-3′ (reverse). The 2^−ΔΔCt^ method was adopted to determine the relative mRNA expression. Each assay was performed in triplicate.

### Western blot assay

EPCs were lysed with RIPA Lysis and Extraction Buffer (Thermo Fisher Scientific, USA) supplemented with protease inhibitor mixture (Thermo Fisher Scientific, USA). Protein concentration was quantified by BCA Protein Assay Kit (Thermo Fisher Scientific, USA). Next, a total of 30 μg proteins were resolved by sodium dodecyl sulfate-polyacrylamide gel electrophoresis (SDS-PAGE). Then, the proteins were transferred to poly vinylidene difluoride membrane (Millipore, USA) at 300 mA for 120 min. After blocked in 5% skimmed milk, the membranes were incubated with primary antibodies against Wnt1 (Abcam, UK) overnight at 4 °C (1:1000). After being washed by PBST, goat-anti-rabbit secondary antibody conjugated with horseradish peroxidase (HRP) (Abcam, UK) was used to bind with primary antibody (1:5000). Finally, protein bands were imaged with Clarity Max Western ECL Substrate (Bio-rad, USA), and quantitative densitometry analysis of protein bands was conducted through Quantity One Software (Bio-rad, USA).

### Mir-326-5p transfection

MiR-326-5p agomir, antagomir, and negative oligonucleotide were synthesized by GenePharma (China). EPCs were digested, manually counted and seeded onto a 6-well plate, then incubated for 24 h at 37 °C, and then transfected with miR-326-5p agomir, antagomir, or scramble control by Lipo 2000 (Invitrogen, USA) as described previously [17].

### EPC tube formation assay

The tube formation assay was conducted to determine the tube incorporation potential of EPCs in vitro. In brief, EPCs were labeled with DiI for 20 min. After being washed with PBS, 2000 of the DiI-labeled cells were mixed with 4.0 × 10^4^ human umbilical cord-derived endothelial cells (HUVECs) in 100 μL of EBM-2, then seeded together on a 96-well plate, with 50 μl Matrigel matrix (BD, USA) added to each well beforehand, and incubated for 30 min at 37 °C. The number of incorporated DiI-labeled cells was counted and averaged with a computer-assisted fluorescent microscope (OLYMPUS, Japan) (Additional file 1).

### Matrigel plug angiogenesis assay

Matrigel plug assays were performed to measure the angiogenesis as previously described [18]. Briefly, EPCs were loaded with miR-326-5p NC/miR-326-5p agomir/miR-326-5p antagomir/miR-326-5p agomir+Wnt1 agonist, respectively. Next, 1 × 10^6^ EPCs were mixed with 500 μl of Matrigel (BD, USA) at a ratio of 1:1. Then, the cell suspensions were injected subcutaneously in the dorsal region of nude mice (female, 7–8 weeks old). After 4 weeks, plugs were removed, photographed, and then embedded in OCT, cryostat sectioned, and examined by the subsequent immunofluorescence assay.

### Acute MI model and assessment of heart function

An acute myocardial infarction (AMI) was generated in mice as described previously [19]. Briefly, C57BL/6 mice (female, 8~10 weeks old) were intubated under general anesthesia (100 mg/kg ketamine, 10 mg/kg xylazine, i.p.) and mechanically ventilated. MI was induced by permanent ligation of the left anterior descending artery (LAD) with a 6-0 suture. PBS (control), EPCs-NC (EPCs were transfected with nonsense sequence), miRNA-326-5p-EPCs (EPCs were transfected with miRNA-326-5p), and miRNA-326-5p-EPCs+ Wnt1 agonist were transplanted (intramyocardial injection) into the peri-infarcted area, respectively, based on the experimental design. The chest was then closed, and the animals were allowed to recover in a warm and clean cage. Fifteen mice were assigned in each group.

Echocardiographic examinations were performed to determine cardiac function at 7 and 28 days after MI. Hearts were viewed in the short-axis between the two papillary muscles, and each measurement was obtained with M-mode by averaging results from three consecutive heartbeats. Left ventricular ejection fraction (LVEF), fractional shortening (FS), left ventricular end systolic diameter (LVESD), and left ventricular end diastolic diameter (LVEDD) were automatically calculated by the echocardiography software according to the Teicholz formula. Finally, the mice were sacrificed to harvest the heart tissue for histological and immunofluorescence analysis.

### Histological and immunofluorescence analysis

Immunofluorescence staining was performed to evaluate the capillary density of the Matrigel plug. Briefly, the sections were blocked with 3% bovine serum albumin (BSA) for 30 min and incubated overnight at 4 °C with the primary antibody against CD31. Secondary antibody goat anti-mouse Alexa 594 (1:500, Life Technologies) was used for detection. Images were obtained using a fluorescence microscope (OLYMPUS, Japan). The numbers of capillaries in Matrigel plugs were counted and averaged in each group.

All mice were euthanized by cervical dislocation at 28 days after surgery as described previously [20]. Heart tissue was fixed in 4% paraformaldehyde (PFA) and then embedded in OCT and cut into 4-μm-thick slices. Masson’s trichrome was performed to evaluate the fibrosis of myocardial infarction area. The infarcted area was measured using Image J software 1.40 (National Institutes of Health, Bethesda, MD, USA), and the size of the infarction area was expressed as a percentage of the whole left ventricle area.

To detect the capillary density of the peri-infarcted area, 1 mg/mL *Bandeiraea simplicifolia* lectin 1 (BS1 lectin, Vector, USA) was directly injected into the LV chamber through cardiac puncture before MI-induced mice were euthanized. After dewaxing, the sections were stained with goat anti-BS1 lectin antibody (1:100, Vector, USA), and then washed with PBS, and stained with Alexa Fluor 594 rabbit anti-goat IgG (1:500, Invitrogen, USA) at room temperature for 1 h. To detect the arteriole density of peri-infarcted area, we stained the sections with anti-α-smooth muscle actin (α-SMA) antibody (1:250, Dako, USA), washed them with PBS, and stained them with Alexa Fluor 594 donkey anti-mouse IgG 2a (1:1000, Invitrogen, USA) at room temperature for 1 h. Images were acquired using a fluorescence microscope (OLYMPUS, Japan). The numbers of capillaries and arterioles in the peri-infarcted area were counted and averaged in each group.

### Statistical analysis

All results are expressed as mean ± SD. The data were analyzed using GraphPad Prism 6.0 software. Two-tailed Student’s *t* test was used for comparing two independent groups. Comparisons of multiple independent groups were analyzed using one-way analysis of variance (ANOVA) followed by a Student-Newman-Keuls test. Differences were considered significant at *P* < 0.05.

## Results

### Identification and characterization of EPCs

**Fig. 1:**
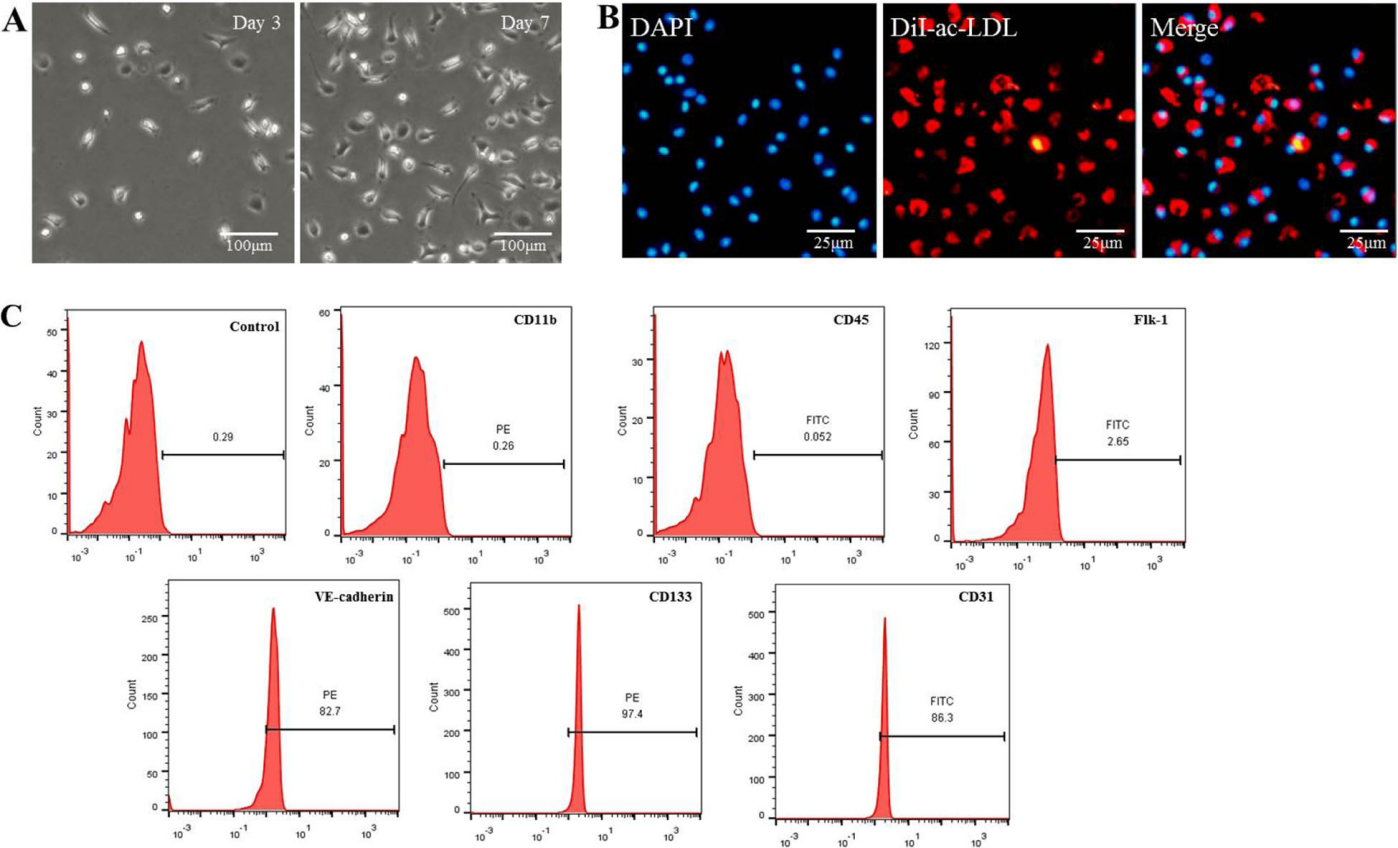
Characterization of endothelial progenitor cells (EPCs). **a** Morphology of EPCs was observed under a microscope. Bar, 100 μm. **b** DiI-ac-LDL (red) could be taken up by EPCs (the nucleus stained with DAPI, blue) as visualized through immunofluorescence staining. Bar, 25 μm. **c** Immunophenotypic analyses of EPC surface markers by flow cytometry

EPCs were isolated from mouse bone marrow. Adherent cells changed their morphology to spindle-shape after 3 days and then formed typical colonies after 7 days (Fig. 1a). As a unique characteristic, EPCs could uptake Dil-ac-LDL as previously reported (Fig. 1b). The surface markers of EPCs were further identified through flow cytometry, which were positive for CD31, CD133, and VE-cadherin and negative for CD11b, Flk-1, and CD45 (Fig. 1c).

### Wnt1 is a target gene of miR-326-5p

**Fig. 2:**
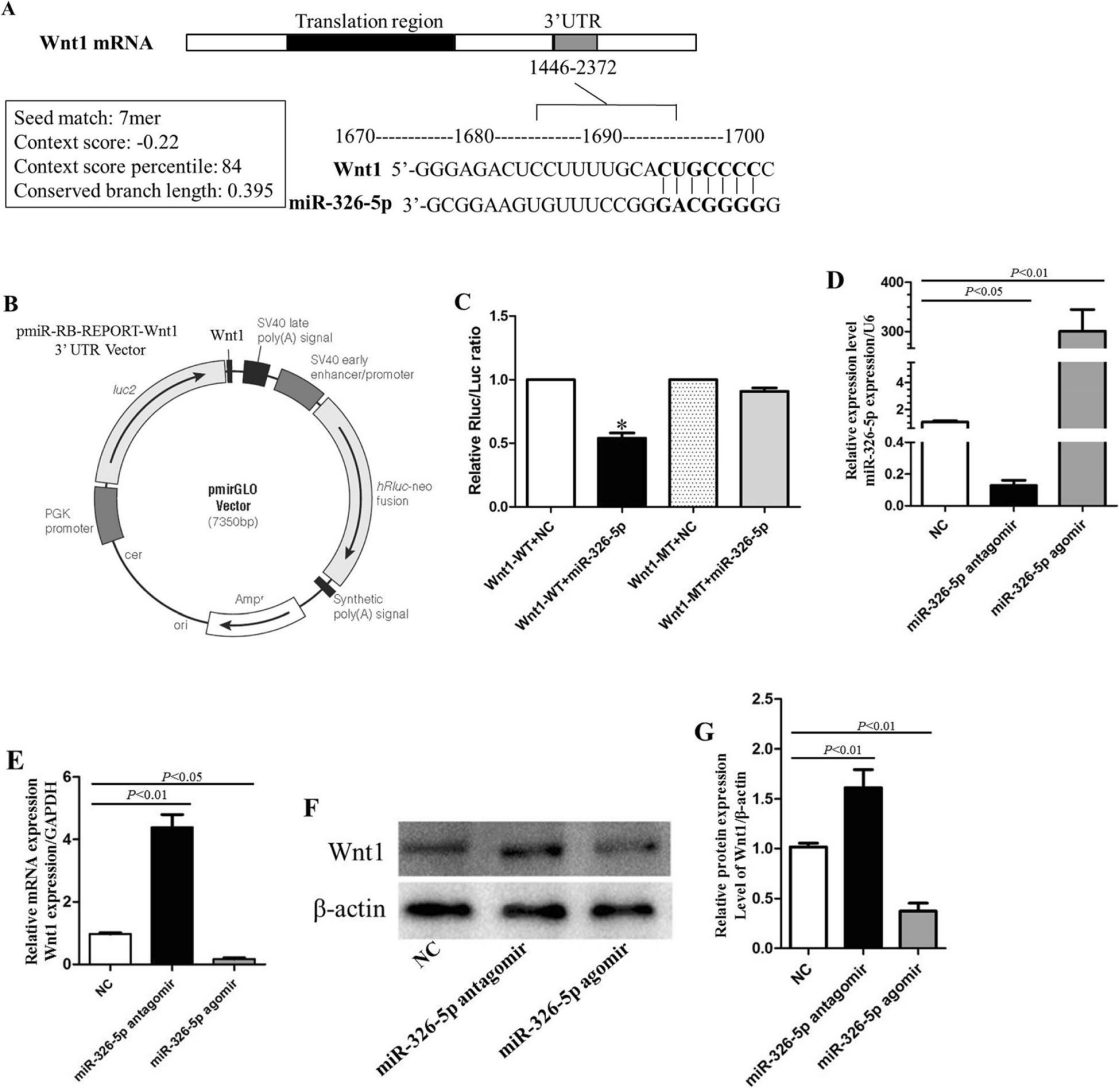
Wnt1 is a putative target of miR-326-5p in EPCs. **a** Binding motif of miR-326-5p on position 243–249 of Wnt1 3′UTR. **b** Luciferase of the pmirGLO vector containing Wnt1 gene was constructed. **c** Relative Rluc/Luc ratio (*n* = 3). *P < 0.01, Wnt1-WT+miR-326-5p vs. Wnt1-WT+NC. **d** The expression level of miR-326-5p was determined by RT-qPCR in three groups (NC group: EPCs; miR-326-5p antagomir group: EPCs transfected with miR-326-5p antagomir; miR-326-5p agomir group: EPCs transfected with miR-326-5p agomir; *n* = 3, respectively). **e** mRNA expression of Wnt1 in the NC group, miR-326-5p antagomir group, and miR-326-5p agomir group (*n* = 3). **f** Protein expression of Wnt1 was detected by Western blot in the NC group, miR-326-5p antagomir group, and miR-326-5p agomir group (*n* = 3). **g** Qualification of Wnt1 protein expression level (*n* = 3). The *P* values for comparison were indicated in the images, respectively

Three databases (TargetScan, microRNA, and mirWALK) were used to screen the putative target genes of miR-326-5p. Wnt1 was one of the potential target genes. miR-326-5p was predicted to be able to bind to the 3′UTR of Wnt1 (Fig. 2a). To further investigate the interaction between miR-326-5p and Wnt1, we constructed the luciferase reporter vector (Fig. 2b) and performed the luciferase reporter assay. As shown in Fig. 2c, miR-326-5p remarkably decreased the relative activity of luciferase reporter of the wild-type Wnt1 3′-UTR, whereas that of the mutant Wnt1 3′-UTR was not significantly changed, which suggested that miR-326-5p could directly bind to the 3′-UTR of Wnt1.

Furthermore, we quantified the Wnt1 expression level in EPCs transfected with miR-326-5p agomir or miR-326-5p antagomir through RT-qPCR. The expression of miR-326-5p was significantly upregulated and downregulated after transfection with agomir and antagomir respectively (Fig. 2d), suggesting that the cells were successfully transfected. Meanwhile, the overexpression of miR-326-5p in EPCs was accompanied by the decreased expression of Wnt1 mRNA, and vice versa (Fig. 2e). Consistently, the expression level of Wnt1 protein was also reduced, and the reduction was negatively correlated with the expression level of miR-326-5p (Fig. 2f, g). Together, these results indicated that Wnt1 was a putative target of miR-326-5p.

### Incorporation ability of EPCs into tube structure was enhanced by transfection with miR-326-5p in vitro

**Fig. 3:**
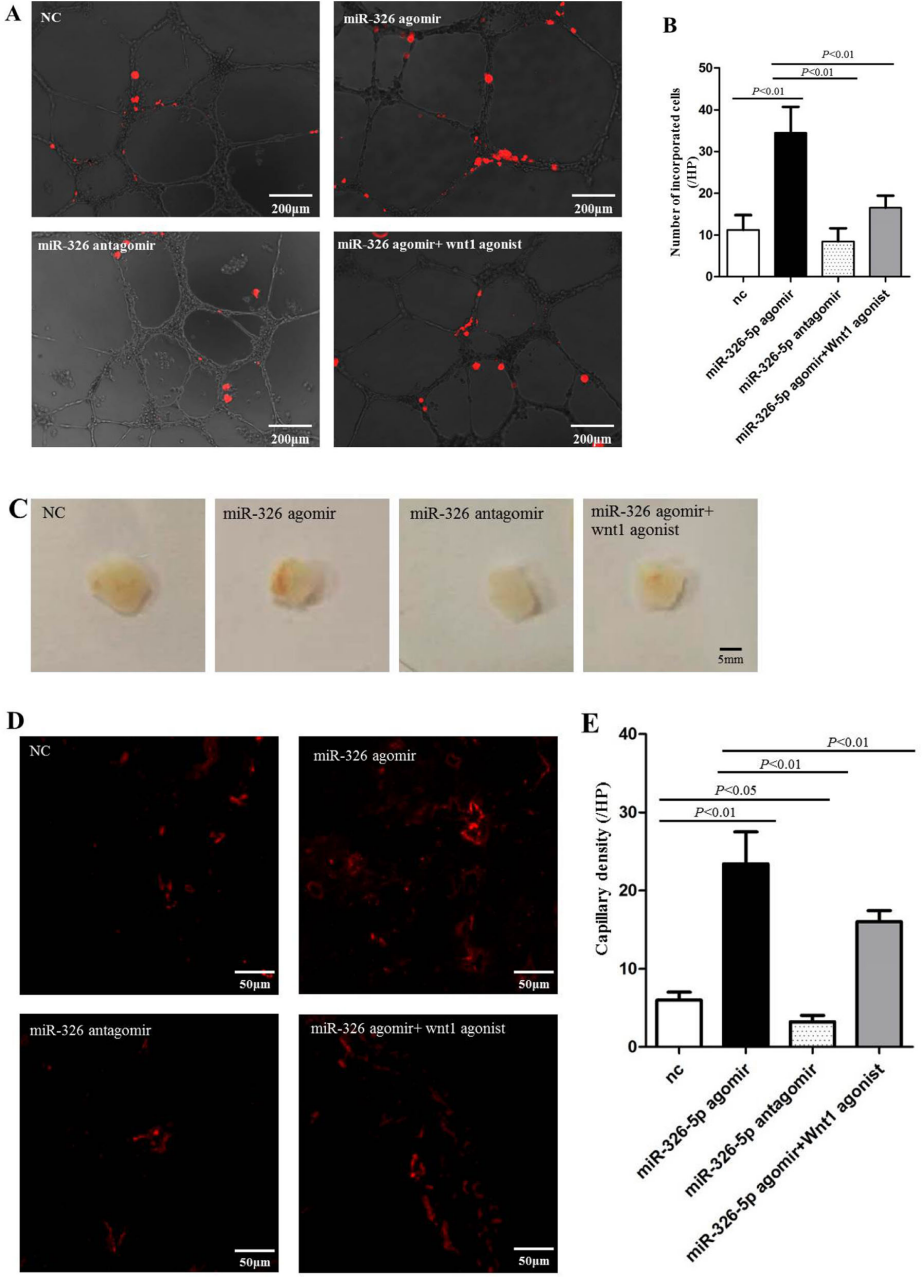
miR-326-5p promoted EPC angiogenesis in vitro and in vivo. **a** Tube formation assay on Matrigel was assessed 6 h after seeding HUVECs pretreated with DiI-labeled EPCs/NC, miR-326-5p agomir EPCs, miR-326-5p antagomir EPCs, or miR-326-5p agomir EPCs+ Wnt1 agonist. DiI-positive incorporated EPCs were observed in the HUVEC-formed tube structure (red). Bar, 200 μm. **b** The number of DiI-positive incorporated EPCs was counted and averaged in each group (*n* = 4). **c** Gross look of Matrigel plugs. Bar, 5 mm. **d** Capillaries of immunofluorescence were observed (red, bar 50 μm) in the sections of Matrigel plugs and **e** were counted and averaged in each group (*n* = 5). The *P* values for comparison were indicated in the images, respectively

To determine whether miR-326-5p could enhance the angiogenic behavior of EPCs, we carried out the tube formation assay using Matrigel in vitro. We found that DiI-labeled EPCs (red) incorporated into the tube structure formed by HUVECs (Fig. 3a). In fact, the number of labeled EPCs in miR-326-5p agomir group was significantly increased (Fig. 3b), compared with those in the negative control (NC) group and miR-326-5p antagomir group (*P* < 0.01, miR-326-5p agomir 34.5 ± 6.2/HPF vs. NC 11.3 ± 3.5/HPF and miR-326-5p antagomir 8.5 ± 3.1/HPF), whereas the number was significantly reduced by the treatment of Wnt1 agonist (*P* < 0.01, miR-326-5p agomir vs. miR-326-5p agomir+Wnt1 agonist). However, transfection of miR-326-5p antagomir could not significantly reduce the incorporated numbers of EPCs compared with that in the NC group. This assay revealed that overexpressing miR-326-5p in EPCs was closely related to the angiogenic capacity of the cells.

### Angiogenic capacity of EPCs was promoted by transfection with miR-326-5p in vivo

Through the subcutaneous Matrigel plug assay, we further examined the angiogenic effect of miR-326-5p on EPCs in vivo. Matrigel containing EPCs (NC group), EPCs transfected with miR-326-5p agomir (miR-326-5p agomir group), miR-326-5p antagomir (miR-326-5p antagomir group), or miR-326-5p agomir+Wnt1 agonist (miR-326-5p agomir+Wnt1 agonist group), was injected subcutaneously into male mice in the inguinal regions respectively. After 14 days, Matrigel was excised, and subsequently, the presence of blood vessels was assessed by immunofluorescence staining of CD31 (red). Matrigel plug in the miR-326-5p agomir group showed much more vessels than other groups (Fig. 3c). Moreover, the morphology and number of vessels in Matrigel plugs were directly visualized by immunofluorescence staining (Fig. 3d). Consistent with in vitro data, the number of vessels in the miR-326-5p agomir group was significantly increased, compared with that in the NC group and miR-326-5p antagomir group (*P* < 0.01, miR-326-5p agomir 23.4 ± 4.1/HPF vs. NC 6.0 ± 1.0/HPF and miR-326-5p antagomir 3.2 ± 0.8/HPF), whereas the number was significantly reduced by the Wnt1 agonist (*P* < 0.01, miR-326-5p agomir vs. miR-326-5p agomir+Wnt1 agonist) (Fig. 3e). Meanwhile, transfection of miR-326-5p antagomir could obviously reduce the number of vessels in Matrigel plugs compared with that in the NC group (miR-326-5p antagomir vs. NC, *P* = 0.0117). This assay further indicated that miR-326-5p prompted the angiogenic capacity of EPCs.

### The transplantation of miR-326-5p-transfected EPCs effectively improved cardiac function and reduced fibrosis

**Fig. 4:**
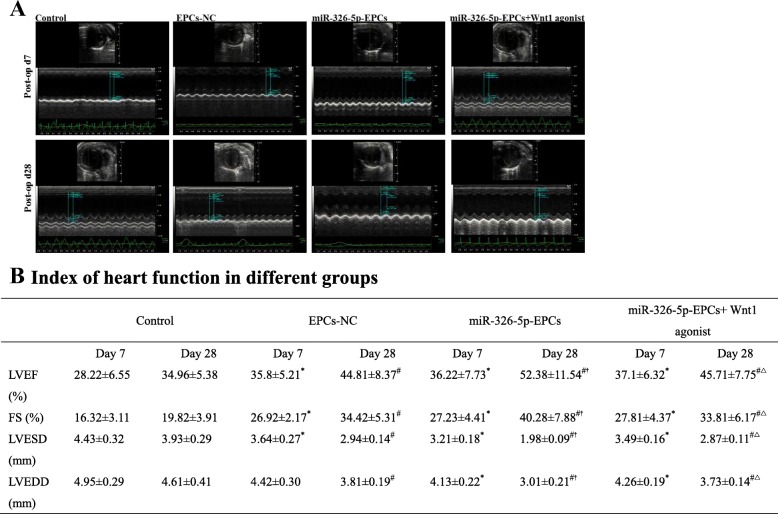
Analysis of mouse cardiac function by echocardiography. **a** Echocardiography was measured at 7 and 28 days postoperatively in the control, EPCs-NC, miR-326-5p-EPCs, and miR-326-5p-EPCs+ Wnt1 agonist groups. **b** Index of heart function in different groups (*n* = 15 in each group). *P < 0.05 compared with the control group at 7 days, ^#^*P* < 0.05 compared with the control group at 28 days, ^†^*P* < 0.05 compared with the EPCs-NC group at 28 days, ^△^*P* < 0.05 compared with the miR-326-5p-EPCs group at 28 days. EF, ejection fraction; FS, fractional shortening; LVESD, left ventricular end systolic diameter; LVEDD, left ventricular end diastolic diameter

To evaluate the cardiac function, we performed echocardiography at 7 and 28 days after MI. The index of heart function in different groups is presented in Fig.4a, b. At 7 days postoperatively, both LVEF and FS were significantly enhanced in the EPCs-NC, miR-326-5p-EPCs, and miR-326-5p-EPCs+ Wnt1 agonist groups, compared with the control group respectively (EPCs-NC vs. control, *P* < 0.05, respectively; miR-326-5p-EPCs vs. control, *P* < 0.05, respectively; miR-326-5p-EPCs+ Wnt1 agonist vs. control, *P* < 0.05, respectively). Meanwhile, LVESD and LVEDD were remarkably decreased in the miR-326-5p-EPCs group and miR-326-5p-EPCs+ Wnt1 agonist group compared with the control group (*P* < 0.05, respectively). However, LVEF, FS, LVESD, and LVEDD remained the same among the EPCs-NC group, miR-326-5p-EPCs group, and miR-326-5p-EPCs+ Wnt1 agonist group. At 28 days after MI surgery, LVEF and FS were remarkably augmented in the miR-326-5p-EPCs group compared with the EPCs-NC and control groups (*P* < 0.05, respectively). Meanwhile, LVESD and LVEDD were also significantly decreased in the miR-326-5p-EPCs group compared with the EPCs-NC and control groups (*P* < 0.05, respectively). Nevertheless, LVEF and FS were significantly decreased in the miR-326-5p-EPCs+ Wnt1 agonist group, accompanied by the remarkable dilatation of LVESD and LVEDD compared with the miR-326-5p-EPCs group (*P* < 0.05, respectively).

**Fig. 5:**
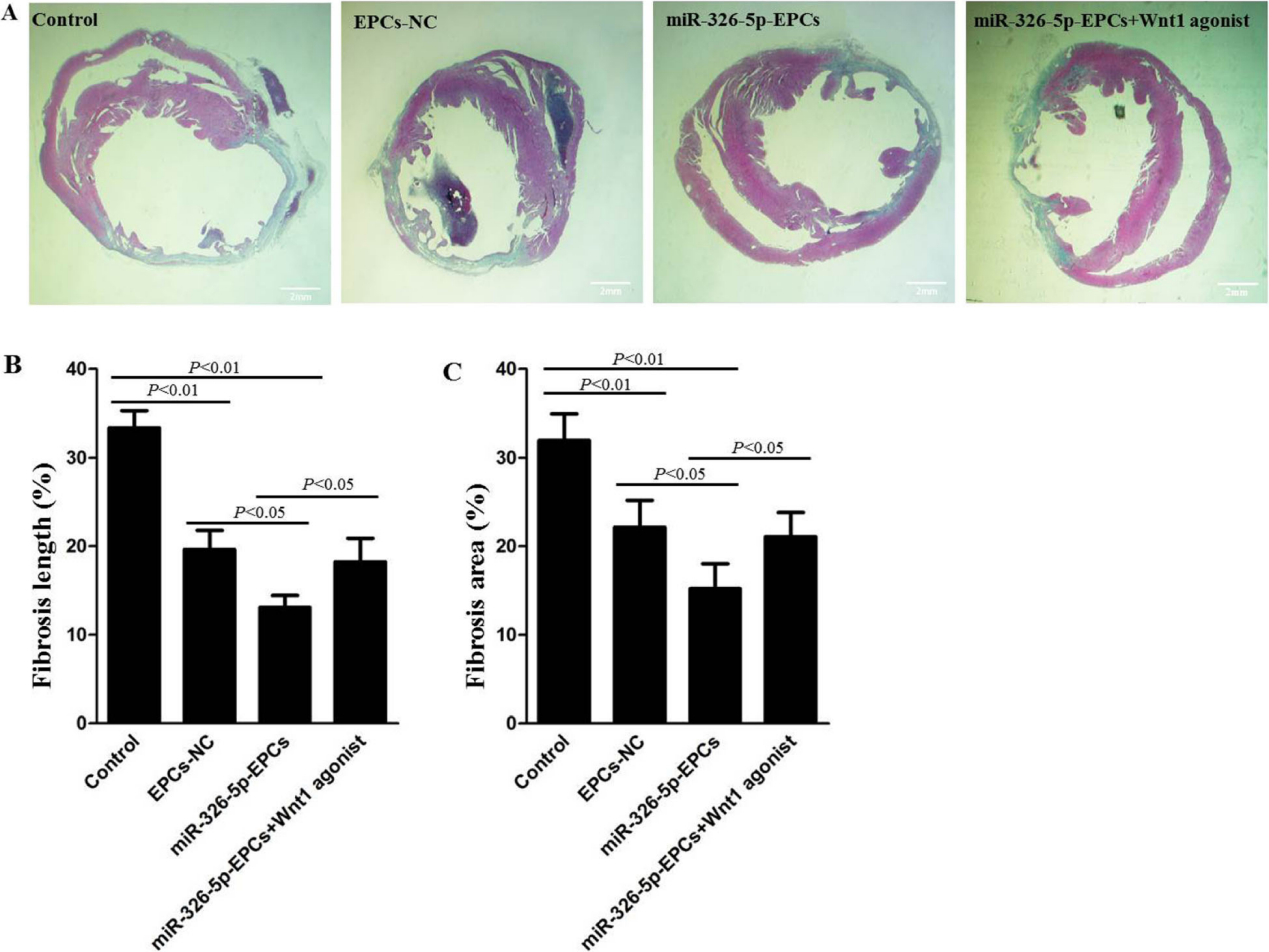
Histological analysis of myocardial infarction (MI) sizes in each group. After MI was inducted, miRNA-326-5p-EPCs (EPCs were transfected with miRNA-326-5p), miRNA-326-5p-EPCs+ Wnt1 agonist, EPCs-NC, and PBS control were injected into the peri-infarct zones. Heart samples in each group were harvested at 28 days after the operation. **a** Cardiac fibrosis was indicated by staining with Masson’s trichrome: scarred fibrosis (blue), myocardium (red). Bar, 2 μm. The percentage of fibrosis length (**b**) and fibrotic area (**c**) were calculated and averaged by using Image J software

To investigate the size of MI, Masson’s trichrome staining was performed at 28 days postoperatively. As shown in Fig. 5a, the scarred fibrosis (blue) and normal myocardium (red) were stained in heart sections. Using Image J software for quantification, we found that the percentage of fibrotic length in the entire internal LV circumference and the percentage of fibrotic area in the entire LV cross-sectional area were both significantly decreased in the miR-326-5p-EPCs group compared with the control and EPCs-NC groups (*P* < 0.01, miR-326-5p-EPCs vs. control, respectively; *P* < 0.05, miR-326-5p-EPCs vs. EPCs-NC, respectively) (Fig. 5b, c). In addition, these values in the EPCs-NC group were also significantly decreased compared with the control group (*P* < 0.01, EPCs-NC vs. control, respectively). However, the percentage of fibrotic length and the percentage of fibrotic area were dramatically increased in the miR-326-5p-EPCs+ Wnt1 agonist group compared with the miR-326-5p-EPCs group (*P* < 0.05, respectively).

### Enhanced neovascularization in ischemic hearts by the transplantation of miR-326-5p-transfected EPCs

**Fig. 6:**
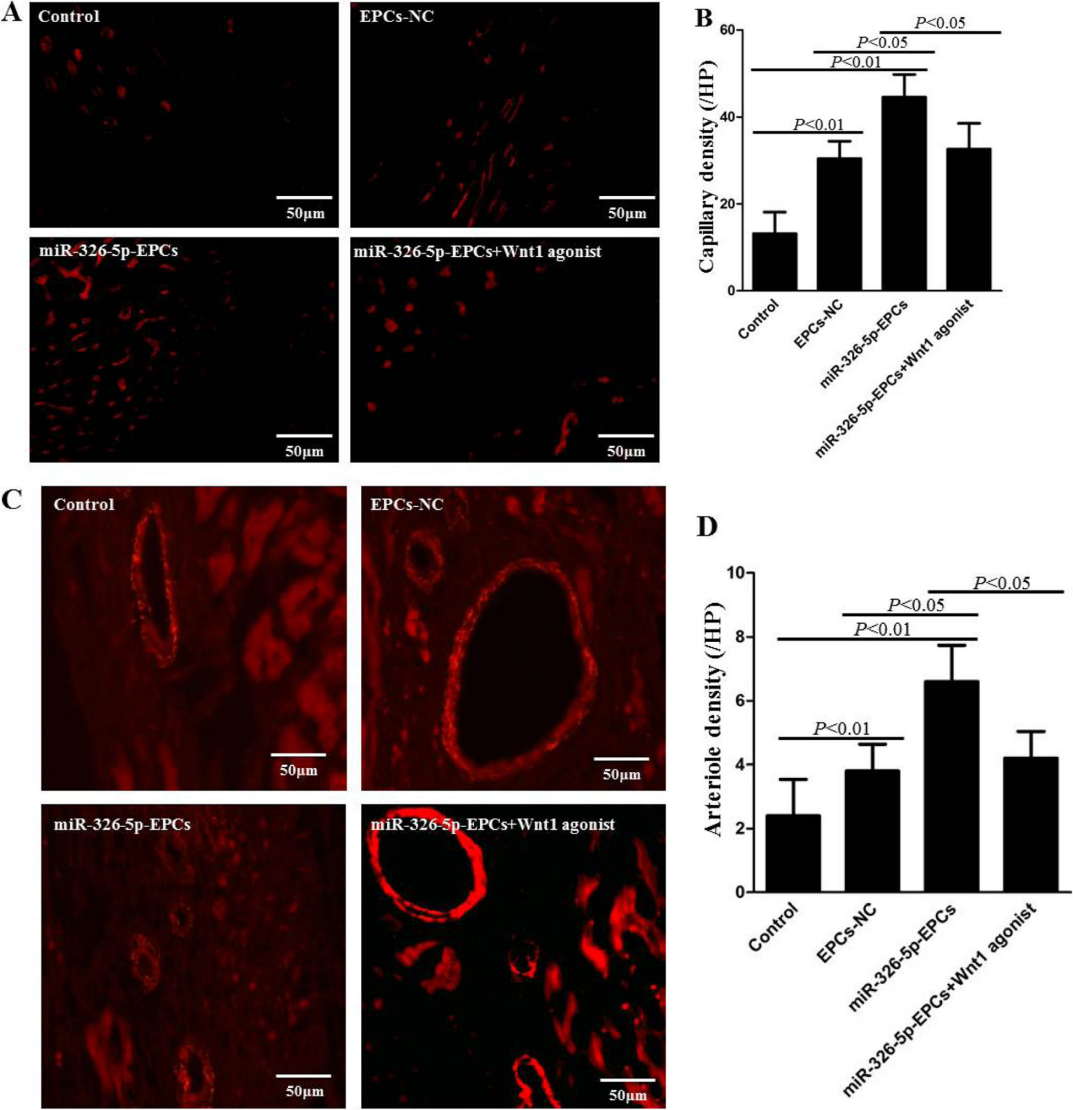
Histological analyses of arteriole density and capillary density in the peri-infarct myocardium. After MI was inducted, miRNA-326-5p-EPCs (EPCs were transfected with miRNA-326-5p), miRNA-326-5p-EPCs+ Wnt1 agonist, EPCs-NC, and PBS (control) were injected into the peri-infarct zones. Heart samples in each group were harvested at 28 days after the operation and then were systemically perfused by BS1 lectin. **a** Capillaries were observed as tubular structure (red) in the peri-infarcted zone. Bar, 50 μm. **b** Capillary density was indicated by counting the numbers of the tubular structure and averaged in each group. **c** Arterioles were visualized by staining heart sections with anti-α-smooth muscle actin(α-SMA) antibody (red) in the peri-infarct zone. Bar, 50 μm. **d** The numbers of arterioles were counted and averaged in each group. The *P* values for comparison were indicated in the images, respectively

To detect the capillaries in the peri-infarcted area, BS1 lectin was injected into the LV chamber using cardiac puncture before euthanizing the mouse. The capillaries were visualized through immunofluorescence staining of BS1 lectin (Fig. 6a, red), and the number of capillaries was counted per HPF and averaged in each group (Fig. 6b). The capillary density in the miR-326-5p-EPCs group was greatly increased compared with the control and EPCs-NC groups (miR-326-5p-EPCs 46.6 ± 6.7, EPCs-NC 36.2 ± 9.4, control 13.2 ± 4.9; *P* < 0.01, miR-326-5p-EPCs vs. control; *P* < 0.05, miR-326-5p-EPCs vs. EPCs-NC). Also, the capillary density in the EPCs-NC group was greatly increased compared with the control group (*P* < 0.01, EPCs-NC vs. control). However, the capillary density was dramatically decreased in the miR-326-5p-EPCs+ Wnt1 agonist group compared with the miR-326-5p-EPCs group (*P* < 0.05).

In addition, the average numbers of arterioles per HPF were also detected through immunofluorescence staining of α-SMA (red) in Fig. 6c. As shown in Fig. 6d, the arteriole density in the miR-326-5p-EPCs group was remarkably enhanced compared with the control and EPCs-NC groups (miR-326-5p-EPCs 17.0 ± 4.0, EPCs-NC 10.8 ± 2.8, control 5.6 ± 2.4; *P* < 0.01, miR-326-5p-EPCs vs. control; *P* < 0.05, miR-326-5p-EPCs vs. EPCs-NC). Moreover, the arteriole density in the EPCs-NC group was also significantly increased compared with the control group (*P* < 0.01, EPCs-NC vs. control). However, the arteriole density was dramatically decreased in the miR-326-5p-EPCs+ Wnt1 agonist group compared with the miR-326-5p-EPCs group (*P* < 0.05). These results demonstrated that miR-326-5p-overexpressing EPCs could enhance neovascularization through targeting Wnt1 in ischemic hearts, consistent with the in vitro data.

## Discussion

In this study, we first confirmed that overexpressing miR-326-5p in EPCs remarkably increased the capacity of tube formation and angiogenesis in the Matrigel plug. Furthermore, miR-326-5p-overexpressing EPCs significantly promoted angiogenesis in the peri-MI region and improved cardiac function in an animal model of AMI. Mechanistically, miR-326-5p facilitated angiogenesis by downregulating the expression level of its target gene Wnt1.

The main pathophysiological change of AMI is local ischemia and oxygen deficit. Therefore, the induction of revascularization is one key therapeutic approach for the stem cell-based therapy of IHD. However, previous evidence demonstrated that transplantation of stem cells alone may be insufficient for revascularization [21]. Hence, epigenetic methods were proposed to modify stem cells, in order to augment the capacity of angiogenesis and cardiac repair. MicroRNAs, a category of small noncoding RNAs, are able to promote both physiological and pathological angiogenesis by negatively regulating protein expression [22, 23]. Researchers have confirmed that microRNA-modified stem cells could promote angiogenesis, augment oxygen supply, reduce myocardial fibrosis, and improve cardiac function [15].

Therefore, we selected several miRNA candidates that had been shown to hold the potential of regulatory angiogenesis. Tube formation analysis demonstrated that miR-326-5p were particularly potent in EPCs compared with control. EPCs were transfected with miR-326-5p agomir and antagomir for gain- and loss-of-function investigation. miR-326-5p agomir greatly promoted the number of EPCs incorporated into HUVEC-formed tube and angiogenesis in Matrigel plugs. These findings illustrated that miR-326-5p played a critical role in EPCs for angiogenesis. Kim’s lab [24] also found that miR-326 could regulate the tumor-induced angiogenesis through targeting HDAC3, and there was a negative feedback loop between HDAC3 and miR-326.

Next, we further confirmed that miR-326-5p-overexpressing EPCs for therapeutic MI significantly enhanced the capillary and arteriole density in the peri-MI region compared with PBS and EPCs-NC groups. Probably attributed to the enhancement of angiogenesis, the LVEF and FS were also significantly elevated at 28 days postoperatively in the miR-326-5p-EPCs group, accompanied by the remarkable reduction of cardiac fibrosis, compared with the PBS and EPCs-NC groups. However, the LVEF and FS between the EPCs-NC group and miR-326-5p-EPCs group at 7 days postoperatively did not show a significant statistical difference. We speculated that 7 days may be too short for miR-326-5p in EPCs to come into play for myocardial repair. Although neovascularization was significantly increased after EPC treatment, we found that the engraftment of EPCs was extremely rare in or around the myocardial infarction area at 28 days after transplantation. We speculated that it might be through the paracrine effect to improve the local microenvironment and promote the regeneration of new capillaries. Meanwhile, as for the treatment of ischemic heart disease with the transplantation of adult stem cells, the mainstream view is that the paracrine effect, rather than direct differentiation, is mainly responsible for the repair of damaged myocardium and improvement of cardiac function [25, 26].

Furthermore, we investigated the potential mRNA targets of miR-326-5p through an online database. Using luciferase reporter assay, RT-qPCR, and Western blot, we identified many mRNA candidates related to vascular formation and finally found that Wnt1 had significantly regulatory changes. Functional experiments showed that recombinant Wnt1 protein could compromise the effect of miR-326-5p on the angiogenesis in vivo and tube incorporation ability of EPCs in vitro. Meanwhile, the enhanced capacity of neovascularization of miR-326-5p-overexpressing EPCs for therapeutic MI was significantly neutralized by recombinant Wnt1 protein, accompanied by the decreased improvement in cardiac function in vivo. These findings demonstrate that miR-326-5p plays a role in angiogenesis of EPCs through targeting Wnt1. Reis et al. [27] reported that Wnt1 could lead to the inhibition of angiogenesis in glioma, which indicated that Wnt1 expression was negatively correlated with angiogenesis. We checked the effect of Wnt-1 agonist alone on angiogenesis in vitro (Additional file 1: Figure S1). Tube formation assay on Matrigel was assessed 6h after seeding HUVECs treated with Wnt-1 agonist. We also found that Wnt1 was negatively correlated with angiogenesis. And this is in line with our results that angiogenesis was facilitated by miR-326-5p agomir through targeting Wnt1.

## Conclusions

In conclusion, our research demonstrated for the first time that miR-326-5p could significantly enhance the angiogenic capacity of EPCs and promote functional cardiac repair of EPCs in an acute MI model, including improvement of LVEF and reduction of myocardial fibrosis, probably by enhancing angiogenesis.

## Supplementary information

**Additional file 1: Figure S1.** The effect of Wnt-1 agonist alone on angiogenesis in vitro*.* (A) Tube formation assay on Matrigel was assessed 6 h after seeding HUVECs treated with Wnt-1 agonist (0 nM, 50 nM, 100 nM). (B) Tube length was measured and compared to NC (*n* = 5/group). **Figure S2.** The expression level of miR-326-5p and Wnt-1 mRNA in the peri-infarcted region. After the injection of miR-326-5p-EPCs, relative expression level of miR-326-5p and Wnt-1 mRNA in the peri-infarcted region was measured compared with negative control using QT-qPCR at 1, 3, 7, 14, 28 days. (A) Relative expression level of miR-326-5p. ^*^P < 0.01, compared with NC group; ^#^*P* < 0.05, compared with NC group. (B) Relative mRNA expression level of Wnt-1/GAPDH. ^*^P < 0.01, compared with NC group; ^#^*P* < 0.05, compared with NC group.

## Data Availability

All data generated or analyzed during this study are included in this published article.

## Abbreviations

AMI: Acute myocardial infarction
BM-MNCs: Bone marrow-mononuclear cells
BS1 lectin: *Bandeiraea simplicifolia* lectin 1
BSA: Bovine serum albumin
EPCs: Endothelial progenitor cells
FBS: Fetal bovine serum
HRP: Horseradish peroxidase
HUVECs: Human umbilical cord-derived endothelial cells
IHD: Ischemic heart diseases
LAD: Left anterior descending artery
LVEF: Left ventricular ejection fraction
miR: MicroRNAs
NC: Negative control
PFA: Paraformaldehyde
SDS-PAGE: Sodium dodecyl sulfate-polyacrylamide gel electrophoresis
STEMI: ST-segment-elevation myocardial infarction
VCAM-1: Vascular cell adhesion molecule
α-SMA: α-Smooth muscle actin
