# Supplementary material for: MicroRNA-326-5p enhances therapeutic potential of endothelial progenitor cells for myocardial infarction
Source: Stem Cell Res Ther. 2019 Nov 15;10:323. doi: 10.1186/s13287-019-1413-8 (PMC6858781; doi:10.1186/s13287-019-1413-8)

figure S1. The effect of Wnt-1 agonist alone on angiogenesis *in vitro.*

(A) Tube formation assay on Matrigel was assessed 6h after seeding HUVECs treated with Wnt-1 agonist (0nM, 50nM, 100nM). (B) Tube length was measured and compared to NC (n=5/group).


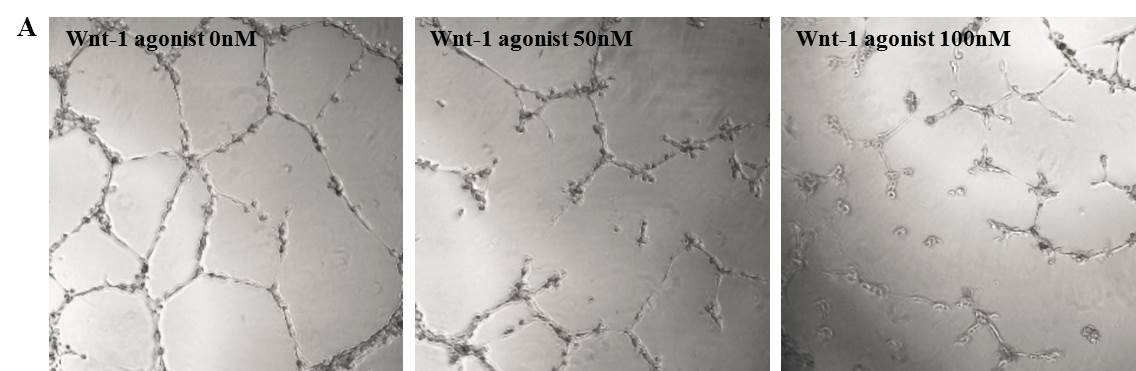

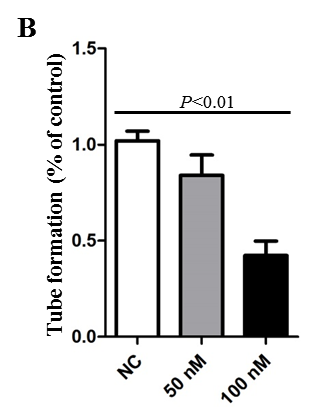


figure S2. The expression level of miR-326-5p and Wnt-1 mRNA in the peri-infarcted region.

After the injection of miR-326-5p-EPCs, relative expression level of miR-326-5p and Wnt-1 mRNA in the peri-infarcted region was measured compared with negative control using QT-qPCR at 1, 3, 7, 14, 28 days. (A) Relative expression level of miR-326-5p. ^*^*P*<0.01, compared with NC group; ^#^*P*<0.05, compared with NC group. (B) Relative mRNA expression level of Wnt-1/GAPDH. ^*^*P*<0.01, compared with NC group; ^#^*P*<0.05, compared with NC group.


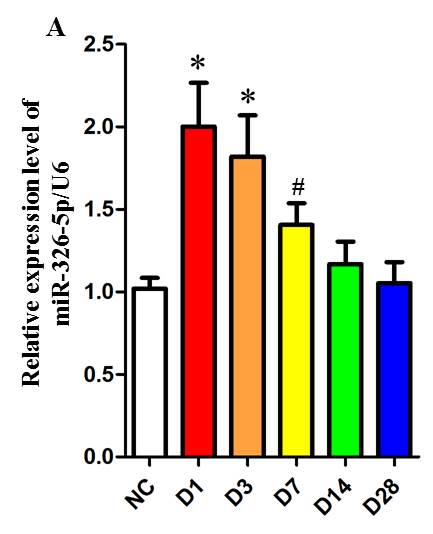

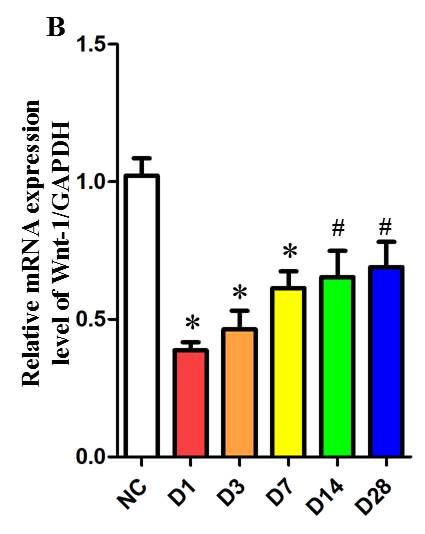

Supplement: Supplementary file 1 — Additional file 1: Figure S1. The effect of Wnt-1 agonist alone on angiogenesis in vitro. (A) Tube formation assay on Matrigel was assessed 6 h after seeding HUVECs treated with Wnt-1 agonist (0 nM, 50 nM, 100 nM). (B) Tube length was measured and compared to NC (n = 5/group). Figure S2. The expression level of miR-326-5p and Wnt-1 mRNA in the peri-infarcted region. After the injection of miR-326-5p-EPCs, relative expression level of miR-326-5p and Wnt-1 mRNA in the peri-infarcted region was measured compared with negative control using QT-qPCR at 1, 3, 7, 14, 28 days. (A) Relative expression level of miR-326-5p. *P < 0.01, compared with NC group; #P < 0.05, compared with NC group. (B) Relative mRNA expression level of Wnt-1/GAPDH. *P < 0.01, compared with NC group; #P < 0.05, compared with NC group. [file 13287_2019_1413_MOESM1_ESM.docx]
